# The Signaling Mechanism of Remote Postconditioning of the Heart: Prospects of the Use of Remote Postconditioning for the Treatment of Acute Myocardial Infarction

**DOI:** 10.3390/cells12121622

**Published:** 2023-06-14

**Authors:** Vyacheslav V. Ryabov, Evgenii V. Vyshlov, Leonid N. Maslov, Alexandr V. Mukhomedzyanov, Natalia V. Naryzhnaya, Alla A. Boshchenko, Aleksandra E. Gombozhapova, Julia O. Samoylova

**Affiliations:** Cardiology Research Institute, Tomsk National Research Medical Center of the RAS, 634012 Tomsk, Russia; rvvt@cardio-tomsk.ru (V.V.R.); evv@cardio-tomsk.ru (E.V.V.); sasha_m91@mail.ru (A.V.M.); natalynar@yandex.ru (N.V.N.); bosh@cardio-tomsk.ru (A.A.B.); gombozhapova@gmail.com (A.E.G.); samoylova.ssmu@yandex.ru (J.O.S.)

**Keywords:** heart, remote postconditioning, ischemia, reperfusion, kinases, end-effector

## Abstract

Acute myocardial infarction (AMI) remains the leading cause of mortality in the world, highlighting an urgent need for the development of novel, more effective approaches for the treatment of AMI. Remote postconditioning (RPost) of the heart could be a useful approach. It was demonstrated that RPost triggers infarct size reduction, improves contractile function of the heart in reperfusion, mitigates apoptosis, and stimulates autophagy in animals with coronary artery occlusion and reperfusion. Endogenous opioid peptides and adenosine could be involved in RPost. It was found that kinases and NO-synthase participate in RPost. KATP channels, MPT pore, and STAT3 could be hypothetical end-effectors of RPost. Metabolic syndrome and old age abolish the cardioprotective effect of RPost in rats. The data on the efficacy of RPost in clinical practice are inconsistent. These data are discussed in the review.

## 1. Introduction

In-hospital mortality in patients with ST-segment elevation myocardial infarction (STEMI) is 5–7% and has not decreased in recent years [1,2,3]. The only effective treatment for acute myocardial infarction (AMI) is recanalization of infarct-related coronary arteries by thrombolysis or percutaneous coronary intervention (PCI). In recent years, the effectiveness of reperfusion therapy has reached its maximum, so in-hospital mortality is not decreasing [1,2,3].

Reperfusion injury to the heart is becoming increasingly relevant in cardiac injury as a result of the increased efficiency of recanalization of infarct-related arteries [4]. There is a clear urgent need to develop new approaches to prevent cardiac reperfusion injury. Remote postconditioning (RPost) could be such an approach. Remote postconditioning is the increased tolerance of an organ to reperfusion after long-term ischemia as a result of the short-term ischemia/reperfusion (I/R) of a remote organ.

The RPost phenomenon was discovered by Kerendi et al. in 2005 [5]. We believe that a study of the molecular mechanisms of RPost could serve as a basis for the development of fundamental novel drugs that can prevent reperfusion cardiac injury. The main manifestations of RPost are a reduction in infarct size [5,6,7], apoptosis inhibition [8,9,10,11], an improvement in the contractility of the heart [12,13,14], the stimulation of autophagy [11,13], and oxidative stress inhibition [9,15].

## 2. Experimental Data

### 2.1. The Involvement of the Nervous System in the Mechanism of Remote Postconditioning

Rats underwent coronary artery occlusion (CAO, 30 min) and reperfusion (2 h) [16]. RPost was mimicked by vagal stimulation. Stimulation of n. vagus decreased the infarct size [16]. In another study, rats were subjected to CAO (30 min) and reperfusion (2 h) [6]. RPost was triggered by a 15 min occlusion of femoral arteries. RP induced infarct size reduction by 56%. Vagotomy or the denervation of hind limbs completely abolished the cardioprotection triggered by RPost. Investigators concluded that the peripheral nervous system is involved in the cardioprotective effect of RPost [6]. However, sensory nerves probably did not participate in RPost because the activation of the transient receptor potential cation channel-1 (TRPV1 channel) localized on sensor nerves by capsaicin 5 min before reperfusion did not affect infarct size [6]. Mice underwent CAO (45 min) and reperfusion (24 h) [17]. RPost was mimicked by a 2 cm transverse incision to the abdominal midline at the end of CAO. Nociceptive RPost caused a decrease in infarct size by 72%. Investigators concluded that skin nociceptors could be involved in trauma-induced cardioprotection [17]. These findings are questionable because the thoracotomy that precedes the CAO is itself a strong trauma. In addition, animals were anesthetized by pentobarbital which contributed to a decrease in pain sensitivity. Mice underwent CAO (45 min) and reperfusion (4 or 24 h) [18]. Nociception was induced during reperfusion by electrical stimulation of needles inserted into the skeletal muscle. Lidocaine was injected subcutaneously to block skin sensory nerves, Nociceptive postconditioning reduced infarct size by about 60%. Lidocaine eliminated nociceptive postconditioning. The infarct-sparing effect of nociceptive-induced postconditioning was not found in TRPV1-channel knockout mice [18]. These findings indirectly indicate that the nervous system and sensory nerves could be involved in RPost. However, the significance of the results was questionable because investigators used small groups of mice (n = 6) [18]. Rats were subjected to CAO (30 min) and reperfusion (3 h) [19]. RPost was performed by three cycles of the occlusion (5 min) and reperfusion (5 min) of both femoral arteries using clamps immediately after the onset of reperfusion. It was found the TRPV1 channel blocker capsazepine completely reversed the infarct-limiting effect of RPost in rats with CAO (30 min) and reperfusion [19]. CGRP8-37, a calcitonin gene-related peptide (CGRP) receptor antagonist (2 mg/kg intravenously, 2 min before reperfusion), also abolished RPost-induced cardiac tolerance to reperfusion. RPost increased the CGRP level in the heart and plasma [19]. Consequently, the TRPV1 channel and CGRP are involved in RPost-triggered cardioprotection. We tried evaluating the role of the autonomic nervous system in RPost. Rats were subjected to CAO (45 min) and reperfusion (2 h) [20]. RPost was induced by the repeated I/R of hindlimbs. Intravenous administration of the nicotinic receptor blocker hexamethonium (10 mg/kg) for 5 min completely reversed the infarct-limiting effect of RPost. Hexamethonium is the ganglion blocker. Hexamethonium itself had no effect on infarct size in non-adapted rats. The number of animals in each group was 12. Hexamethonium completely abolished the infarct-reducing effect of RPost. We proposed that these findings demonstrated the involvement of the autonomic nervous system in the cardioprotective effect of RPost. However, another possibility exists. It was reported that the selective nicotinic α7nAChR receptor agonist PNU282987 can mimic RPost in rats with CAO (30 min) and reperfusion (120 min) [21]. It is possible that hexamethonium blocked the α7nAChR receptor in cardiomyocytes and the autonomic nervous system is not involved in the infarct-sparing effect of RPost.

**Figure 1:**
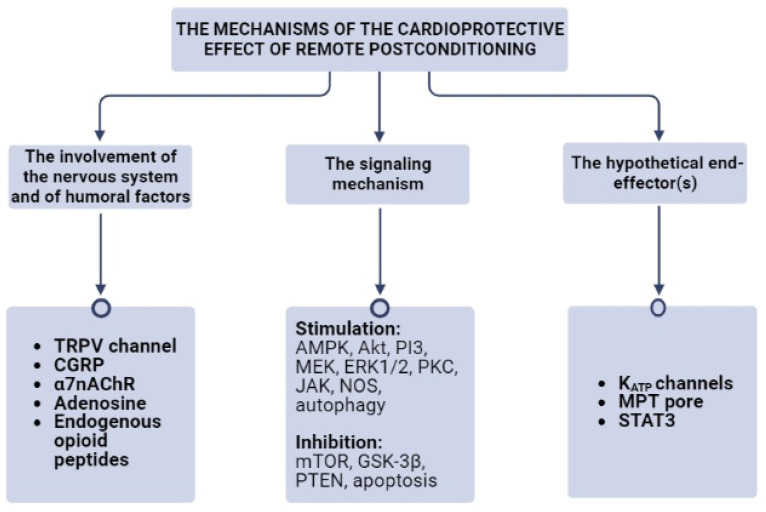
The mechanisms of the cardioprotective effect of remote postconditioning. α7nAChR, the alpha-7 nicotinic receptor; Akt, protein kinase B; AMPK, AMP activated protein kinase; CGRP, the calcitonin gene-related peptide; ERK1/2, extracellular regulated kinase; GSK-3β, glycogen synthase kinase-3β; JAK, janus kinase; K_ATP_ channels, ATP-sensitive K^+^ channels; MEK, mitogen-activated protein kinase kinase; MPT pore, mitochondrial permeability transition pore; mTOR, mammalian target of rapamycin; NOS, NO-synthase; PI3, phosphoinositide-3-kinase; PKC, protein kinase C; PTEN, phosphatase and tensin homolog; STAT3, signal transducer and activator of transcription 3; TRPV, receptor potential cation channel.

These findings demonstrated that the nervous system, the autonomic nervous system, the TRPV1 channel, CGRP, and α7nAChR receptor could be involved in the cardioprotective effect of RPost (Figure 1, Table 1).

### 2.2. The Involvement of Humoral Factors in the Mechanism of Remote Postconditioning

Rats were subjected to CAO (30 min) and reperfusion (180 min) [5]. RPost was induced by renal artery occlusion for 5 min and reperfusion before the restoration of coronary blood flow. The non-selective adenosine receptor antagonist 8-sulfophenyl theophylline (8-SPT, 10 mg/kg) was injected 5 min before reperfusion. It was found that RPost triggered a decrease in infarct size by about 50%. This effect was completely reversed by 8-SPT. Permanent renal artery occlusion had no effect on infarct size [5]. These data demonstrate that some cardioprotective substance is released from the kidney and protects the heart against reperfusion. Investigators did not detect adenosine levels in blood [5]. It was reported that the half-life of adenosine in blood is 10 s [22]. Therefore, signal transmission from kidneys to the heart via adenosine is questionable. It is probably more likely that adenosine acts on the heart’s level. However, the possibility that RPost induced the long-term release of adenosine from the kidney cannot be ruled out.

The isolated rat heart was subjected to three cycles of global ischemia (3 min) and reperfusion (5 min) [23]. The coronary effluent was collected during reperfusion. The isolated rat heart was subjected to CAO (30 min) and reperfusion (120 min), and the heart was perfused by the coronary effluent after the onset of reperfusion three times for 30 s (total 90 s). This impact caused a decrease in infarct size by about 50% [23]. Investigators could isolate the hydrophobic fraction of the coronary effluent which can mimic RPost. This fraction contained proteins with a molecular weight < 10 kDa [23].

Hydrophobic peptide molecules can penetrate the blood–brain barrier [24]. It was reported that RPost can protect the brain against I/R [25,26,27]. These data indirectly confirm the hydrophobic nature of molecules involved in RPost.

**Table 1:** The involvement of nervous system and humoral factors in remote postconditioning of the heart.

RPost Type	Effect	Animals	NS/HF	Reference
Trauma-induced RPost	IS ↓	mice	NS	[17]
Pain electrical stimulation	IS ↓	mice	NS	[18]
Femoral artery O/R	IS ↓	rats	NS, TRPV1	[19]
Hindlimb I/R	IS ↓	rats	NS, AG	[20]
Renal artery O/R	IS ↓	rats	HF, adenosine	[5]
I/R of the heart	IS ↓	rats, CE	HF, peptide	[23]
Hindlimb I/R	IS ↓	pigs	HF	[28]
Trauma-induced RPost	IS ↓	mice	HF, Bradykinin	[17]
Hindlimb I/R	IS ↓	rats	HF, OP	[20]

AG, autonomic ganglion; CE, coronary effluent; HF, humoral factor; IS, infarct size; NS, nervous system; O/R, occlusion/reperfusion; OP, opioid peptide; RPost, remote postconditioning; TRPV1, transient receptor potential cation channel-1; I/R, ischemia/reperfusion.

RPost was induced by ischemia (four cycles for 5 min) and reperfusion (three cycles for 5 min) of the hindlimb in pigs [28]. The plasma of postconditioned pigs was used for perfusion of the isolated rat heart subjected to global ischemia (30 min) and reperfusion (120 min). Pig plasma reduced the infarct size by 34% [28]. This evidence demonstrates the involvement of non-identified humoral factor(s) in RPost-induced cardioprotection.

Mice underwent CAO (45 min) and reperfusion (2 h) [17]. RPost was mimicked by an abdominal incision in mice anesthetized by pentobarbital. Administration of the bradykinin-2 receptor antagonist Hoe 140 completely abolished cardioprotection induced by a painful stimulus. Bradykinin-2 receptor knockout also eliminated the infarct-reduced effect of nociceptive postconditioning. The β-adrenergic receptor blocker propranolol (2 mg/kg intravenously) also reversed nociceptive postconditioning [17]. This study induced many questions and doubts about the significance of the result. Propranolol itself can mimic RPost at a dose of 0.5 mg/kg intravenously [29]. The β-adrenergic receptor antagonist nadolol can also mimic RPost [29]. CAO is accompanied by severe chest trauma and the stimulation of pain receptors. It is unclear why this trauma does not protect the heart against I/R.

We found that blocking peripheral opioid receptors completely abolished the infarct-reducing effect of RPost induced by hind limb I/R in rats [20]. It was detected that opioid peptide deltorphin II can mimic RPost [30,31].

In 2016, Koyama et al. found that the intracoronary administration of Ringer’s solution containing lactate did not aggravate I/R cardiac injury in patients with STEMI and PCI [32]. Investigators proposed that lactate can mimic RPost [33,34].

Recently, it has been demonstrated that exogenous lactate can mimic RPost in rats [35]. It is possible that lactate released from skeletal muscle can trigger RPost in limb I/R.

These data indicate that endogenous opioid peptides, CGRP, lactate, and adenosine could be involved in RPost (Figure 1). Non-identified hydrophobic peptide molecules can also participate in RPost. The involvement of bradykinin or catecholamines in RPost needs to be re-examined (Table 1).

## 3. The Signaling Mechanism of Remote Postconditioning

It was reported that kinases are involved in ischemic preconditioning and postconditioning [36,37], and are also involved in RPost [38].

### 3.1. AMPK and mTOR

It was reported that AMP-activated protein kinase (AMPK) participates in RPost [11]. Rats underwent permanent CAO. RPost was performed by three cycles of left forelimb ischemia (5 min) and reperfusion (5 min), and was repeated every day for 3 days. RPost caused a decrease in infarct size by about 30% and inhibited apoptotic cell death in the heart. RPost reduced the phosphorylated mammalian target of rapamycin (p-mTOR) level [11]. This kinase is an endogenous inhibitor of autophagy [39]. It was found that RPost resulted in an increase in the marker levels of autophagy in myocardial tissue (Beclin-1 and LC3 II), and caused an increase in their p-AMPK content. Pretreatment with the AMPK inhibitor compound C abolished the infarct-limiting effect of RPost [11].

Consequently, RPost stimulates AMPK and autophagy, and inhibits mTOR and apoptosis (Figure 1).

### 3.2. Akt, ERK, and PI3 Kinase

RPost was performed by three cycles of right hindlimb I/R in rats with CAO (45 min) and reperfusion (180 min) [40]. RPost decreased infarct size by 35% and increased p-Akt levels in the myocardium. Wortmannin, a phosphatidylinositol 3-kinase (PI3-kinase) inhibitor, reversed the increase in p-Akt content in myocardial tissue [40]. RPost was induced by four cycles of ischemia (5 min) and reperfusion (5 min) in pigs [28], and reduced infarct size by 33%. The plasma of postconditioned pigs was used for perfusion of the isolated rat heart subjected to global ischemia (30 min) and reperfusion (120 min). Pig plasma reduced infarct size by 34% [28]. This cardioprotective effect of pig plasma was abolished by wortmannin, a phosphatidylinositol 3-kinase (PI3-kinase) inhibitor, and U0126, a mitogen-activated protein kinase (MEK) inhibitor. U0126 simultaneously inhibits the extracellular signal-regulated kinase-1/2 (ERK1/2)-localized downstream of MEK [28]. It was reported that RPost induced by three cycles of hindlimb ischemia (5 min) and reperfusion (5 min) resulted in an increase in the p-Akt level in murine myocardium [41]. Mice were subjected to CAO (45 min) and reperfusion (120 min) [14]. RPost was carried out by three cycles of unilateral hindlimb ischemia (5 min) and reperfusion (5 min). It decreased infarct size by about 30%, and increased the p-Akt kinase level in myocardial tissue. The PI3-kinase inhibitor LY294002 abolished an increase in the p-Akt kinase level. Rats were subjected to CAO (45 min) and reperfusion (120 min) [7]. RPost was performed by three cycles of ischemia (5 min) and reperfusion (5 min) of both hindlimbs, and reduced infarct size by about 50%. The PI3-kinase inhibitor wortmannin abolished the infarct-limiting effect of RPost [7].

Thus, PI3-kinase, Akt, MEK, ERK1/2 are involved in RPost (Figure 1).

### 3.3. Protein Kinase C (PKC)

It was found that the PKC inhibitor chelerythrine completely reversed the infarct-reducing effect of RPost triggered by three cycles of bilateral hindlimb I/R in rats with CAO (45 min) and reperfusion (120 min) [7]. Consequently, PKC is involved in RPost (Figure 1).

### 3.4. JAK

Mice underwent CAO (45 min) and reperfusion (120 min) [41]. RPost was carried out by three cycles of ischemia (5 min) and reperfusion (5 min) of the left hind limb, and it limited infarct size by 44% and inhibited apoptosis. The Janus kinase (JAK) inhibitor AG490 reversed the infarct-reducing effect RPost [41]. Thus, JAK participates in RPost (Figure 1).

### 3.5. JNK and GSK-3β

The inhibition of glycogen synthase kinase-3β (GSK-3β) or c-Jun N-terminal kinase (JNK) promoted an increase in cardiac tolerance to I/R [42,43]. Rats underwent CAO (30 min) and reperfusion (120 min) [21]. RPost was performed by bilateral hind limb ischemia (10 min) and following reperfusion, and limited infarct size by about 19%. RPost stimulated the phosphorylation (inactivation) of GSK-3β [21], and increased the p-GSK-3β levels in myocardial tissue in mice [44]. Limb RPost reduced infarct size, the serum cTroponin I, and creatine kinase-MB levels in rats [21]. This cardioprotective effect was associated with an increase in phosphorylated GSK-3β content in myocardial tissue.

Thus, GSK-3β inhibition is involved in RPost (Figure 1). The role of JNK inhibition in the cardioprotective effect of RPost has not been studied before.

### 3.6. PTEN

Phosphatase and Tensin Homolog (PTEN) catalyzes kinase dephosphorylation [45]. Mice were subjected to CAO (40 min) and reperfusion (24 h) [43]. RPost was induced by four cycles of occlusion (5 min) and reperfusion (5 min) of the femoral artery. RPost limited infarct size by 40.7% and reduced the number of apoptotic cells in myocardial tissue. RPost decreased the PTEN level and increased the p-Akt and p-GSK-3β levels in myocardial tissue [44]. Mice were fed a 2% cholesterol diet for 12 weeks. This diet abolished RPost-induced cardioprotection, reduced p-Akt and p-GSK-3β levels, and increased the PTEN content in myocardial tissue compared to the RPost + CAO group. The PTEN inhibitor bisperoxovanadium (1.0 mg/kg) restored the cardioprotective effect of RPost in mice with a cholesterol diet [44]. It is possible that the activation of PTEN in mice with a cholesterol diet is a reason for the disappearance of RPost-induced cardiac tolerance to I/R.

Consequently, PTEN inhibition could be involved in RPost (Figure 1).

### 3.7. NO-Synthase

NO-synthase (NOS) is involved in the cardioprotective effect of the second window of ischemic preconditioning [46]. Rabbits were subjected to CAO (30 min) and reperfusion (3 h) [9]. RPost was carried out by occlusion (5 min) of the left pulmonary artery following reperfusion (5 min). Investigators did not evaluate infarct size. RPost resulted in a decrease in the plasma concentration of creatine kinase and malondialdehyde (MDA). These findings indicate that RPost can mitigate excessive reactive oxygen species (ROS) production. Pretreatment with the NOS inhibitor L-NAME abolished the cardioprotective effect of RPost [9]. It was found that limb RPost triggered an increase in endothelial NOS expression in myocardial tissue of rabbits [47]. It was reported that RPost induced by three cycles of hindlimb ischemia (5 min) and reperfusion (5 min) resulted in an increase in the p-eNOS level in the murine myocardium [41].

Thus, there is indirect evidence that NOS could be involved in RPost (Figure 1, Table 2). RPost could be limited by excessive ROS production in reperfusion. It was demonstrated that AMPK, mTOR, Akt, PI3K, ERK1/2, PKC, PTEN, GSK-3β, and JAK could be involved in RPost (Table 2).

## 4. The Hypothetical End-Effector(s) of Remote Postconditioning

The transcription factor signal transducer and activator of transcription 3 (STAT3) plays an important role in ischemic preconditioning and postconditioning [37].

It was found that RPost induced by three cycles of hindlimb ischemia (5 min) and reperfusion (5 min) resulted in an increase in the p-STAT level in murine myocardium [41]. Pigs underwent CAO (55 min) and reperfusion (120 min) [48]. RPost was induced by tightening a tourniquet around the left hindlimb, and four cycles of ischemia (5 min) and reperfusion (5 min) was used. RPost limited infarct size by 51% and increased the p-STAT level [48]. It should be noted that both groups of investigators did not use the STAT inhibitor static. Consequently, there is only indirect evidence of the involvement of STAT3 in RPost.

It was reported that ATP-sensitive K^+^ channels (KATP channels), big conductance Ca^2+^ channels (BKCa channels), and mitochondrial permeability transition pore (MPT pore) participate in the infarct-limiting effect of pre- and postconditioning [36,37,49]. Therefore, we proposed that these molecular structures can be also involved in RPost.

Rats underwent CAO (45 min) and reperfusion (120 min). RPost was induced by three cycles of bilateral hindlimb ischemia (5 min) and reperfusion (5 min). RPost limited infarct size by 50% [20]. We found that the KATP channel blocker glibenclamide completely reversed the infarct-sparing effect of RPost [20]. The MPT pore opener atractyloside also abolished the infarct-reducing effect of RPost [20]. The role of BKCa channels in RPost has not been studied before.

Thus, it is possible that KATP channels, the MPT pore, and STAT3 could be end-effectors of RPost (Figure 1).

## 5. Remote Postconditioning and Experimental Metabolic Syndrome

We already reported above that a long-term 2% cholesterol diet reversed the cardioprotective effect of RPost [44]. We induced metabolic syndrome (MS) in rats with a high-carbohydrate high-fat diet (90 days) [50]. Rats were subjected to CAO (45 min) and reperfusion (120 min). RPost was induced by three cycles of bilateral hindlimb ischemia (5 min) and reperfusion (5 min). The infarct-limiting effect of RPost in young (150 days) rats with MS was less than in animals without MS (*p* < 0.001) [48]. RPost did not reduce infarct size in aged rats (540 days) with MS [20]. We found a direct correlation between infarct size and the plasma leptin level (r = 0.85, *p* < 0.014) in rats with RPost and MS [20,51]. It was reported that leptin increased cardiac tolerance to I/R in rats through the activation of the JAK2/STAT3 signaling pathway where JAK is Janus kinase, and STAT is the signal transducer and activator of transcription [52]. It could be proposed that an increase in the endogenous leptin level will enhance cardiac tolerance to I/R but we found that an increase in the plasma leptin concentration is associated with a rise in infarct size [20]. Our results are consistent with Dixon’s data [53]. This group found that leptin had no cardioprotective effect in Zucker obese (fa/fa) rats with MS [53]. The reason for this disappearance of the cardioprotective effect of leptin in rats with MS has not been studied before.

## 6. The Optimal Protocol of Remote Postconditioning

**Figure 2:**
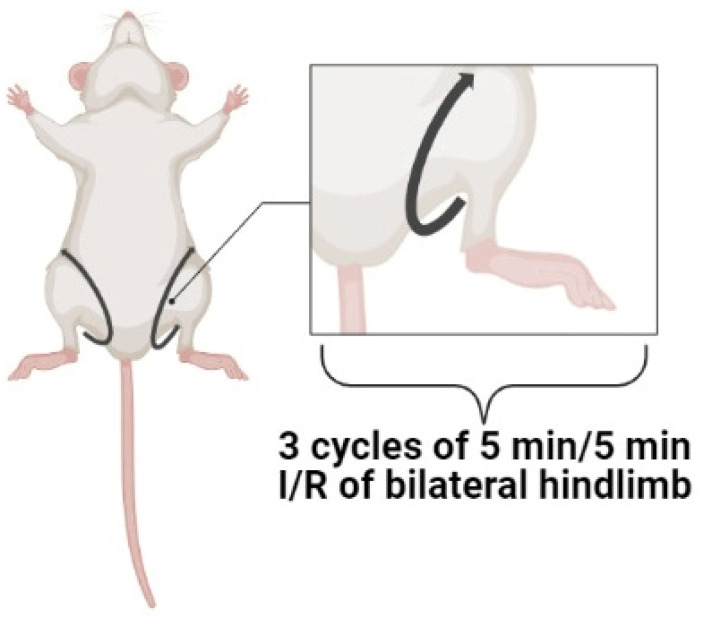
The optimal protocol of remote postconditioning. I/R, ischemia/reperfusion.

Three cycles of bilateral hindlimb I/R promoted infarct size reduction by 36–56% [6,7,19]. Single bilateral hindlimb ischemia contributed to a decrease in infarct size by about 19% [21]. Three or four cycles of unilateral limb I/R promoted infarct size reduction by 30–51% [11,28,41,44,48]. Most investigators used three or four cycles of unilateral hindlimb I/R [28,41,44,48]. Xu et al. (2022) carried out RPost by three cycles of left forelimb ischemia (5 min) and reperfusion (5 min). In this case, RPost caused a decrease of in infarct size by about 30%,

We suggest that the efficiency of RPost depends on the skeletal muscle volume involved in RPost. The larger volume promoted the small infarct size. Single I/R of extremities exhibits a weak infarct-reducing effect. Consequently, three or four cycles of I/R on hindlimbs should be used for an increase in cardiac tolerance to I/R (Figure 2).

## 7. Remote Postconditioning in Clinical Practice

Ninety-six patients with STEMI and PCI were included in the randomized controlled trial [54]. Nine percent of patients had diabetes mellitus. RPost was induced by 3 cycles of 5 min/5 min I/R by cuff inflation/deflation of the lower limb. RPost had no effect on the creatinine kinase-MB (CK-MB) release curve. RPost did not improve contractility of the heart or the incidence of microvascular obstruction (MVO) [54]. RPost was induced in children with open-heart surgery for the repair of congenital heart defects (n = 69) by three cycles of lower limb ischemia (5 min) and reperfusion (5 min) using a blood pressure cuff (200 mmHg) at the onset of aortic unclamping [55]. RPost reduced the postoperative serum cardiac troponin I (cTnI) and CK-MB levels [55].

In a study, 1280 patients were subjected to cardiac surgery [56]. Patients were randomized into the remote preconditioning (RPre) + RPost group or the control group without conditioning. RPost and RPre were triggered by four cycles of ischemia (5 min) and reperfusion (5 min) of the upper limb before cardiopulmonary bypass (CPB) and after CPB. RPre + RPost did not decrease the negative outcome compared with the control group and had no effect on a major adverse outcome. Investigators concluded that RPre + RPost did not improve clinical outcomes in patients subjected to cardiac surgery [56]. Patients with STEMI and PCI (n = 151) underwent the combination of RPer and IPost [57]. RPost and RPre were induced by three cycles of 5-min inflation and 5-min deflation of an upper-arm pressure cuff. The combination of RPer and IPost decreased the serum CK-MB area under the curve over 72 h by 29%, compared to the control group. It was concluded that RPer + IPost reduced infarct size in patients with STEMI and PCI [57]. Forty-six STEMI patients with PCI were included in a study [15]. RPost (n = 23) was triggered by three cycles of ischemia (5 min) and reperfusion (5 min) by cuff inflation/deflation of the lower left limb. It was found that RPost had no effect on infarct size detected by the enzymatic method, but RPost promoted a decrease in the plasma MDA level [15]. The randomized LIPSIA CONDITIONING trial included patients with STEMI (n = 696) and PCI [56]. A combination of RPer and IPost was used in three cycles of a 5-min inflation and a 5-min deflation of the arm [58]. The combination of RPer and IPost had no effect on CK peak, MVO, or ST-segment resolution [58]. In the Remote Ischemic Preconditioning and Postconditioning Outcome (RISPO) trial it was included in cardiac surgery patients (n = 1280) [59]. RPre and Pros were performed by four cycles of inflation (5 min) following deflation (5 min) of a pneumatic cuff around the arm. No differences between groups regarding major adverse cardiac and cerebrovascular events were detected [59]. The single-blind randomized controlled trial CONDI-2/ERIC-PPCI included patients (n = 5115) with STEMI and PCI from 33 centers in European countries [60]. RPost was carried out by four cycles of inflation (5 min) and deflation (5 min) of an automated cuff around the arm. Patients were treated with aspirin, clopidogrel, or ticagrelor orally. High-sensitivity troponin T was used for infarct size measurement. RPost did not improve clinical outcomes (cardiac death or hospitalization for heart failure) at 12 months in patients with STEMI and PCI. RPost had no effect on infarct size. It is unclear whether different or identical kits were used for troponin T measurements. Investigators did not analyze the impact of metabolic syndrome or age on the results of the trial [60]. Therefore, this trial did not answer a number of questions about whether RPost alters the incidence of major adverse cardiovascular events (MACE), infarct size, or microvascular obstruction. It is unknown whether hypercholesterolemia, metabolic syndrome, and old age abolish the cardioprotective effect of RPost, or whether these patients received the KATP channel blocker glibenclamide which abolished the cardioprotective effect of RPost in rats. A study included 270 patients with STEMI and PCI [61]. RPost was performed within 48 h after PCI when reperfusion injury has already occurred [4]. It is possible that for this reason, RPost had no effect on post-infarction remodeling of the heart. A study included patients (n = 70) undergoing on-pump cardiac surgery [62]. RPost was performed by 3 cycles of ischemia (5 min) and reperfusion (5 min) of the arm. It was found that RPost reduced the plasma troponin T and CK-MB levels [62]. These data demonstrated that RPost can mitigate reperfusion cardiac injury.

In summary, the data on the efficacy of RPost in clinical practice are inconsistent (Table 3).

There is evidence that RPost can increase cardiac tolerance to reperfusion [55,57,62]. There are data that indicate that RPost does not affect cardiac tolerance to reperfusion [15,54,56,58,59,60]. Consequently, clinical data are inconsistent. One of the possible reasons for the failure of clinical studies could be the low muscle mass involved in RPost. In addition, the age of patients, the presence of metabolic syndrome or diabetes mellitus, the use of glibenclamide and other KATP channel blockers were not taken into account. It is possible that the duration of ischemia also mattered. The duration of myocardial ischemia was typically 30–60 min in experimental studies. The duration of myocardial ischemia was usually 3–12 h in clinical studies.

## 8. Conclusions

Thus, the main manifestations of RPost are the infarct size reduction, apoptosis inhibition, the improvement of contractility of the heart in reperfusion, the activation of autophagy, and oxidative stress inhibition. It is unknown whether RPost can prevent necroptosis and ferroptosis. There is evidence that the nervous system participates in the infarct-reducing effect of RPost. However, the cardioprotective effect of RPost could be the result of stimulation of the TRPV1 channel in sensory nervous terminals and the α7nAChR receptor activation in the heart. Opioid peptides, CGRP, adenosine, and non-identified hydrophobic peptide could be endogenous humoral factors involved in RPost. AMPK, NOS, PI3-kinase, Akt, MEK, ERK1/2, and JAK are involved in RPost. GSK-3β, mTOR, and PTEN inhibition are also involved in RPost. The role of JNK in the cardioprotective effect of RPost has not been studied before. KATP channels, MPT pore, and STAT3 are hypothetical end-effectors of RPost. The low efficacy of RPost in clinical practice could be due to a number of reasons. RPost in clinical practice is usually induced by ischemia/reperfusion of the arm. RPost in experimental practice is usually induced by ischemia/reperfusion of both hindlimbs. Consequently, greater muscle mass is involved in RPost in animals than in humans. It was used in yang animals without metabolic syndrome and hypercholesterolemia in experimental studies. There are many old patients with metabolic syndrome and hypercholesterolemia patients with AMI. Many patients with AMI receive glibenclamide and other KATP channel blockers which abolish RPost. To resolve the issue of the efficacy of RPost in clinical practice, clinical trials involving non-included old patients and patients with diabetes mellitus and hypercholesterolemia are required. Patients received glibenclamide and other KATP-channel blockers should be excluded from this placebo-controlled trial. Patients with hindlimb cuffing but no I/R should be included in the placebo group. In this case, we obtained data that can be trusted.

## Figures and Tables

**Table 2:** The involvement of kinases and NO-synthase in remote postconditioning of the heart.

Enzyme	RPost Type	Effect	Animals	References
AMPK activation	limb I/R	IS ↓	rats	[11]
mTOR activation	limb I/R	IS ↓	rats	[11]
Akt activation	limb I/R	IS ↓	rats	[40]
PI3K activation	limb I/R	IS ↓	rats	[40]
PI3K activation	limb I/R	IS ↓	pigs	[28]
ERK1/2 activation	limb I/R	IS ↓	pigs	[28]
Akt activation	limb I/R	IS ↓	mice	[14]
PI3K activation	limb I/R	IS ↓	mice	[14]
PI3K activation	limb I/R	IS ↓	rats	[7]
PKC activation	limb I/R	IS ↓	rats	[7]
JAK activation	limb I/R	IS ↓	rats	[41]
GSK-3β inhibition	limb I/R	IS ↓	rats	[21]
GSK-3β inhibition	femoral artery O/R	IS ↓	mice	[44]
PTEN inhibition	femoral artery O/R	IS ↓	mice	[44]
NO-synthase	pulmonary artery O/R	CK ↓	rabbits	[9,47]

Akt, Akt kinase; AMPK, AMP-activated protein kinase; CK, creatine kinase; ERK1/2, extracellular signal-regulated kinase-1/2; GSK-3β, glycogen synthase kinase-3β; I/R, ischemia/reperfusion; JAK, Janus kinase; mTOR, mammalian target of rapamycin; O/R, occlusion/reperfusion; PI3K, phosphatidylinositol 3-kinase; PKC, protein kinase C; PTEN, Phosphatase and Tensin Homolog; RPost, remote postconditioning.

**Table 3:** Application of remote postconditioning in clinical trials.

Groups	Model RPost	Effects	References
96 patients with STEMI and PCI	3 cycles of 5 min/5 min I/R of the lower limb	No effect on CK-MB Did not improve contractility of the heart and the incidence of MVO	[46]
69 children with open-heart surgery	3 cycles of 5 min/5 min I/R of the lower limb	Reduced the postoperative levels of cTnI and CK-MB	[47]
1280 patients with cardiac surgery	4 cycles of 5 min/5 min I/R of the upper limb before CPB and after CPB	Did not improve clinical outcome in patients who underwent cardiac surgery	[48]
151 patients with STEMI and PCI	3 cycles of 5 min/5 min I/R of an upper-arm	Decreased the serum CK-MB within 72 h by 29%	[49]
46 patients with STEMI and PCI	3 cycles of 5 min/5 min I/R of the lower left limb	No effect on infarct size detected by enzymatic method, but a decrease in the plasma MDA level	[15]
696 patients with STEMI and PCI	3 cycles of 5 min/5 min I/R of the arm	No effect on CK-MB peak, MVO, ST-segment resolution	[50]
1280 patients with cardiac surgery	4 cycles of 5 min/5 min I/R of the arm	Not detected differences between groups in major adverse cardiac and cerebrovascular events	[51]
5115 patients with STEMI and PCI	4 cycles of 5 min/5 min I/R of the arm	Did not improve clinical outcomes	[52]
270 patients with STEMI and PCI	RPost was performed within 48 h after PCI	No effect on post-infarction remodeling of the heart	[53,54]
70 patients with cardiac surgery	3 cycles of 5 min/5 min I/R of the arm	Reduced the plasma TnT and CK-MB levels	[55]

CK-MB, the creatinine kinase-MB; cTnI, cardiac troponin I; MDA, malondialdehyde; MVO, microvascular obstruction; PCI, percutaneous coronary intervention; STEMI, ST-segment elevation myocardial infarction; TnT, troponin T.

## Data Availability

No new data were created or analyzed in this study. Data sharing is not applicable to this article.

## References

[1] Menees D.S., Peterson E.D., Wang Y., Curtis J.P., Messenger J.C., Rumsfeld J.S., Gurm H.S. (2013). Door-to-balloon time and mortality among patients undergoing primary PCI. N. Engl. J. Med..

[2] Fabris E., Kilic S., Schellings D.A.A.M., Ten Berg J.M., Kennedy M.W., van Houwelingen K.G., Giannitsis E., Kolkman E., Ottervanger J.P., Hamm C. (2017). Long-term mortality and prehospital tirofiban treatment in patients with ST elevation myocardial infarction. Heart.

[3] Olier I., Sirker A., Hildick-Smith D.J.R., Kinnaird T., Ludman P., de Belder M.A., Baumbach A., Byrne J., Rashid M., Curzen N. (2018). British Cardiovascular Intervention Society and the National Institute for Cardiovascular Outcomes Research. Association of different antiplatelet therapies with mortality after primary percutaneous coronary intervention. Association of different antiplatelet therapies with mortality after primary percutaneous coronary intervention. Heart.

[4] Maslov L.N., Popov S.V., Mukhomedzyanov A.V., Naryzhnaya N.V., Voronkov N.S., Ryabov V.V., Boshchenko A.A., Khaliulin I., Prasad N.R., Fu F. (2022). Reperfusion cardiac injury: Receptors and the signaling mechanisms. Curr. Cardiol. Rev..

[5] Kerendi F., Kin H., Halkos M.E., Jiang R., Zatta A.J., Zhao Z.Q., Guyton R.A., Vinten-Johansen J. (2005). Remote postconditioning. Brief renal ischemia and reperfusion applied before coronary artery reperfusion reduces myocardial infarct size via endogenous activation of adenosine receptors. Basic Res. Cardiol..

[6] Basalay M., Barsukevich V., Mastitskaya S., Mrochek A., Pernow J., Sjöquist P.O., Ackland G.L., Gourine A.V., Gourine A. (2012). Remote ischaemic pre- and delayed postconditioning—Similar degree of cardioprotection but distinct mechanisms. Exp. Physiol..

[7] Mukhomedzyanov A.V., Naryzhnaya N.V., Maslov L.N. (2021). The role of protein kinase C and PI3-kinase in the mechanism of the cardioprotective effect of remote ischemic postconditioning. Bull. Sib. Med..

[8] Ren H.M., Xie R.Q., Cui W., Liu F., Hu H.J., Lu J.C. (2012). Effects of rabbit limbs ischemia/ reperfusion on myocardial necrosis and apoptosis. Zhongguo Ying Yong Sheng Li Xue Za Zhi.

[9] Tang Y.H., Xu J.J., Li J.X., Cheng X.S. (2011). Remote postconditioning induced by brief pulmonary ischemia and reperfusion attenuates myocardial reperfusion injury in rabbits. Chin. Med. J..

[10] Liu S., Wu X.F., Zhang W.Z., Sun Y.X., Cai S.L. (2007). Remote postconditioning by brief renal ischemia and reperfusion reduces acute myocardial ischemia and reperfusion induced myocardial apoptosis in rabbits. Zhonghua Xin Xue Guan Bing Za Zhi.

[11] Xu S., Xia X., Liu Y., Chen F., Gu R., Bian X., Xu X., Jia C., Lu S., Gu Y. (2022). Remote cyclic compression ameliorates myocardial infarction injury in rats via AMPK-dependent pathway. Microvasc. Res..

[12] Nacar A.B., Topcu S., Kurt M., Tanboga I.H., Karakaş M.F., Buyukkaya E., Aksakal E., Sen N., Akcay A.B., Bilen E. (2015). Effect of remote ischemic postconditioning on left ventricular mechanics. Echocardiography.

[13] Han Z., Cao J., Song D., Tian L., Chen K., Wang Y., Gao L., Yin Z., Fan Y., Wang C. (2014). Autophagy is involved in the cardioprotection effect of remote limb ischemic postconditioning on myocardial ischemia/reperfusion injury in normal mice, but not diabetic mice. PLoS ONE.

[14] Wang X., Wang J., Tu T., Iyan Z., Mungun D., Yang Z., Guo Y. (2018). Remote ischemic postconditioning protects against myocardial ischemia-reperfusion injury by inhibition of the RAGE-HMGB1 pathway. Biomed. Res. Int..

[15] Wang N., Wang G.S., Yu H.Y., Mi L., Guo L.J., Gao W. (2014). Myocardial protection of remote ischemic postconditioning during primary percutaneous coronary intervention in patients with acute ST-segment elevation myocardial infarction. Beijing Da Xue Xue Bao.

[16] Wang Q., Cheng Y., Xue F.S., Yuan Y.J., Xiong J., Li R.P., Liao X., Liu J.H. (2012). Postconditioning with vagal stimulation attenuates local and systemic inflammatory responses to myocardial ischemia reperfusion injury in rats. Inflamm. Res..

[17] Song Y., Shan J.G., Xue Z., Wang S.Y., Xu H., Liu Y., Guo Y.S., Ren X. (2016). Remote postconditioning induced by trauma protects the mouse heart against ischemia reperfusion injury. Involvement of the neural pathway and molecular mechanisms. Cardiovasc. Drugs Ther..

[18] Ren X., Roessler A.E., Lynch T.L., Haar L., Mallick F., Lui Y., Tranter M., Ren M.H., Xie W.R., Fan G.C. (2019). Cardioprotection via the skin: Nociceptor-induced conditioning against cardiac MI in the NIC of time. Am. J. Physiol. Heart Circ. Physiol..

[19] Gao Y., Song J., Chen H., Cao C., Lee C. (2015). TRPV1 activation is involved in the cardioprotection of remote limb ischemic postconditioning in ischemia-reperfusion injury rats. Biochem. Biophys. Res. Commun..

[20] Naryzhnaya N.V., Logvinov S.V., Kurbatov B.K., Mukhomedzyanov A.V., Sirotina M.A., Chepelev S.N., Vismont F.I., Maslov L.N. (2022). The efficiency of remote ischemic postconditioning of the myocardium in rats with induced metabolic syndrome depends on the leptin level. Proc. Natl. Acad. Sci. Belarus Med. Ser..

[21] Li H.X., Cui X.L., Xue F.S., Yang G.Z., Liu Y.Y., Liu Q., Liao X. (2018). Inhibition of glycogen synthase kinase-3β is involved in cardioprotection by α7nAChR agonist and limb remote ischemic postconditionings. Biosci. Rep..

[22] Klabunde R.E. (1983). Dipyridamole inhibition of adenosine metabolism in human blood. Eur. J. Pharmacol..

[23] Breivik L., Helgeland E., Aarnes E.K., Mrdalj J., Jonassen A.K. (2011). Remote postconditioning by humoral factors in effluent from ischemic preconditioned rat hearts is mediated via PI3K/Akt-dependent cell-survival signaling at reperfusion. Basic Res. Cardiol..

[24] Maslov L.N., Lishmanov Y.B. (2017). Permeability of the blood-brain barrier for opioid peptides. Exp. Clin. Pharmacol..

[25] Xie B., Gao X., Huang Y., Zhang Y., Zhu S. (2021). Remote Ischemic Postconditioning Inhibits Hippocampal Neuronal Apoptosis and Mitophagy after Cardiopulmonary Resuscitation in Rats. Shock.

[26] Huang Y., Gao X., Zhou X., Zhang Y., Tan Z., Zhu S. (2021). Remote Ischemic Postconditioning Inhibited Mitophagy to Achieve Neuroprotective Effects in the Rat Model of Cardiac Arrest. Neurochem. Res..

[27] Yu H.H., Ma X.T., Ma X., Chen M., Chu Y.H., Wu L.J., Wang W., Qin C., Tian D.S. (2021). Remote Limb Ischemic Postconditioning Protects Against Ischemic Stroke by Promoting Regulatory T Cells Thriving. J. Am. Heart Assoc..

[28] Skyschally A., Gent S., Amanakis G., Schulte C., Kleinbongard P., Heusch G. (2015). Across-species transfer of protection by remote ischemic preconditioning with species-specific myocardial signal transduction by reperfusion injury salvage kinase and survival activating factor enhancement pathways. Circ. Res..

[29] Lishmanov Y.B., Maslov L.N., Mukhomedzyanov A.V. (2016). Role of β-Adrenoceptors and L-Type Ca^2+^-Channels in the Mechanism of Reperfusion-Induced Heart Injury. Bull. Exp. Biol. Med..

[30] Maslov L.N., Mukhomedzyanov A.V., Tsibulnikov S.Y., Suleiman M.S., Khaliulin I., Oeltgen P.R. (2021). Activation of peripheral δ2-opioid receptor prevents reperfusion heart injury. Eur. J. Pharmacol..

[31] Popov S.V., Mukhomedzyanov A.V., Maslov L.N., Naryzhnaya N.V., Kurbatov B.K., Prasad N.R., Singh N., Fu F., Azev V.N. (2023). The Infarct-Reducing Effect of the δ2 Opioid Receptor Agonist Deltorphin II: The Molecular Mechanism. Membranes.

[32] Koyama T., Munakata M., Akima T., Kageyama T., Shibata M., Moritani K., Kanki H., Ishikawa S., Mitamura H. (2016). Impact of postconditioning with lactate-enriched blood on in-hospital outcomes of patients with ST-segment elevation myocardial infarction. Int. J. Cardiol..

[33] Koyama T. (2017). Lactated Ringer’s solution for preventing myocardial reperfusion injury. Int. J. Cardiol. Heart Vasc..

[34] Koyama T., Munakata M., Akima T., Miyamoto K., Kanki H., Ishikawa S. (2019). Muscle squeezing immediately after coronary reperfusion therapy using postconditioning with lactate-enriched blood. Int. J. Cardiol..

[35] Chepelev S.N., Vismont F.I., Goubkin S.V., Maslov L.N. (2021). The influence of old age on cardioprotective efficiency of pharmacological postconditioning using lactic acid in ischemia-reperfusion of the myocardium in experiment. Dokl. Natl. Acad. Sci. Belarus.

[36] Yellon D.M., Downey J.M. (2003). Preconditioning the myocardium: From cellular physiology to clinical cardiology. Physiol. Rev..

[37] de Miranda D.C., de Oliveira Faria G., Hermidorff M.M., Dos Santos Silva F.C., de Assis L.V.M., Isoldi M.C. (2021). Pre- and Post-Conditioning of the Heart: An Overview of Cardioprotective Signaling Pathways. Curr. Vasc. Pharmacol..

[38] Tyagi S., Singh N., Virdi J.K., Jaggi A.S. (2019). Diabetes abolish cardioprotective effects of remote ischemic conditioning: Evidences and possible mechanisms. J. Physiol. Biochem..

[39] Popov S.V., Mukhomedzyanov A.V., Voronkov N.S., Derkachev I.A., Boshchenko A.A., Fu F., Sufianova G.Z., Khlestkina M.S., Maslov L.N. (2022). Regulation of autophagy of the heart in ischemia and reperfusion. Apoptosis.

[40] Yu Y., Jia X.J., Zong Q.F., Zhang G.J., Ye H.W., Hu J., Gao Q., Guan S.D. (2014). Remote ischemic postconditioning protects the heart by upregulating ALDH2 expression levels through the PI3K/Akt signaling pathway. Mol. Med. Rep..

[41] Gao S., Zhan L., Yang Z., Shi R., Li H., Xia Z., Yuan S., Wu Q.P., Wang T., Yao S. (2017). Remote limb ischaemic postconditioning protects against myocardial ischaemia/reperfusion injury in mice: Activation of JAK/STAT3-mediated Nrf2-antioxidant signalling. Cell. Physiol. Biochem..

[42] Miura T., Miki T. (2009). GSK-3β, a therapeutic target for cardiomyocyte protection. Circ. J..

[43] Milano G., Morel S., Bonny C., Samaja M., von Segesser L.K., Nicod P., Vassalli G. (2007). A peptide inhibitor of c-Jun NH_2_-terminal kinase reduces myocardial ischemia-reperfusion injury and infarct size in vivo. Am. J. Physiol. Heart Circ. Physiol..

[44] Hong J., Ge H.W., Liu J.Q., Sun R.H., Kong F.J. (2019). Pharmacological Inhibition of PTEN Restores Remote Ischemic Postconditioning Cardioprotection in Hypercholesterolemic Mice: Potential Role of PTEN/AKT/GSK3β SIGNALS. Shock.

[45] Popov S.V., Maslov L.N., Naryzhnaya N.V., Mukhomezyanov A.V., Krylatov A.V., Tsibulnikov S.Y., Ryabov V.V., Cohen M.V., Downey J.M. (2021). The Role of Pyroptosis in Ischemic and Reperfusion Injury of the Heart. J. Cardiovasc. Pharmacol. Ther..

[46] Baxter G.F., Ferdinandy P. (2001). Delayed preconditioning of myocardium: Current perspectives. Basic Res. Cardiol..

[47] Tang Y.H., Yang J.S., Xiang H.Y., Xu J.J. (2014). PI3K-Akt/eNOS in remote postconditioning induced by brief pulmonary ischemia. Clin. Investig. Med..

[48] Kleinbongard P., Skyschally A., Gent S., Pesch M., Heusch G. (2017). STAT3 as a common signal of ischemic conditioning: A lesson on “rigor and reproducibility” in preclinical studies on cardioprotection. Basic Res. Cardiol..

[49] Behmenburg F., Trefz L., Dorsch M., Ströthoff M., Mathes A., Raupach A., Heinen A., Hollmann M.W., Berger M.M., Huhn R. (2018). Milrinone-induced postconditioning requires activation of mitochondrial Ca^2+^-sensitive potassium (mBKCa) channels. J. Cardiothorac. Vasc. Anesth..

[50] Logvinov S.V., Naryzhnaya N.V., Kurbatov B.K., Gorbunov A.S., Birulina Y.G., Maslov L.N., Oeltgen P.R. (2021). High carbohydrate high fat diet causes arterial hypertension and histological changes in the aortic wall in aged rats: The involvement of connective tissue growth factors and fibronectin. Exp. Gerontol..

[51] Logvinov S.V., Mukhomedzyanov A.V., Kurbatov B.K., Sirotina M.A., Naryzhnaya N.V., Maslov L.N. (2023). Participation of Leptin and Corticosterone in the Decrease in Infarct-Limiting Efficiency of Remote Postconditioning and in the Development of Arterial Hypertension in Metabolic Syndrome in Rats. Bull. Exp. Biol. Med..

[52] Smith C.C., Dixon R.A., Wynne A.M., Theodorou L., Ong S.G., Subrayan S., Davidson S.M., Hausenloy D.J., Yellon D.M. (2010). Leptin-induced cardioprotection involves JAK/STAT signaling that may be linked to the mitochondrial permeability transition pore. Am. J. Physiol. Heart Circ. Physiol..

[53] Dixon R.A., Davidson S.M., Wynne A.M., Yellon D.M., Smith C.C. (2009). The cardioprotective actions of leptin are lost in the Zucker obese (fa/fa) rat. J. Cardiovasc. Pharmacol..

[54] Crimi G., Pica S., Raineri C., Bramucci E., De Ferrari G.M., Klersy C., Ferlini M., Marinoni B., Repetto A., Romeo M. (2013). Remote ischemic post-conditioning of the lower limb during primary percutaneous coronary intervention safely reduces enzymatic infarct size in anterior myocardial infarction: A randomized controlled trial. JACC Cardiovasc. Interv..

[55] Zhong H., Gao Z., Chen M., Zhao J., Wang F., Li L., Dong H., Liu L., Wang Q., Xiong L. (2013). Cardioprotective effect of remote ischemic postconditioning on children undergoing cardiac surgery: A randomized controlled trial. Paediatr. Anaesth..

[56] Hong D.M., Lee E.H., Kim H.J., Min J.J., Chin J.H., Choi D.K., Bahk J.H., Sim J.Y., Choi I.C., Jeon Y. (2014). Does remote ischaemic preconditioning with postconditioning improve clinical outcomes of patients undergoing cardiac surgery? Remote Ischaemic Preconditioning with Postconditioning Outcome Trial. Eur. Heart J..

[57] Prunier F., Angoulvant D., Saint Etienne C., Vermes E., Gilard M., Piot C., Roubille F., Elbaz M., Ovize M., Bière L. (2014). The RIPOST-MI study, assessing remote ischemic perconditioning alone or in combination with local ischemic postconditioning in ST-segment elevation myocardial infarction. Basic Res. Cardiol..

[58] Eitel I., Stiermaier T., Rommel K.P., Fuernau G., Sandri M., Mangner N., Linke A., Erbs S., Lurz P., Boudriot E. (2015). Cardioprotection by combined intrahospital remote ischaemic perconditioning and postconditioning in ST-elevation myocardial infarction: The randomized LIPSIA CONDITIONING trial. Eur. Heart J..

[59] Cho Y.J., Lee E.H., Lee K., Kim T.K., Hong D.M., Chin J.H., Choi D.K., Bahk J.H., Sim J.Y., Choi I.C. (2017). Long-term clinical outcomes of Remote Ischemic Preconditioning and Postconditioning Outcome (RISPO) trial in patients undergoing cardiac surgery. Int. J. Cardiol..

[60] Hausenloy D.J., Kharbanda R.K., Møller U.K., Ramlall M., Aarøe J., Butler R., Bulluck H., Clayton T., Dana A., Dodd M. (2019). CONDI-2/ERIC-PPCI Investigators. Effect of remote ischaemic conditioning on clinical outcomes in patients with acute myocardial infarction (CONDI-2/ERIC-PPCI): A single-blind randomised controlled trial. Lancet.

[61] Ikonomidis I., Vlastos D., Andreadou I., Gazouli M., Efentakis P., Varoudi M., Makavos G., Kapelouzou A., Lekakis J., Parissis J. (2021). Vascular conditioning prevents adverse left ventricular remodelling after acute myocardial infarction: A randomised remote conditioning study. Basic Res. Cardiol..

[62] Garcia-de-la-Asuncion J., Moreno T., Duca A., García-Del-Olmo N., Perez-Griera J., Belda J., Soro M., García-Del-Olmo E. (2022). Effects of remote ischemic postconditioning on HIF-1α and other markers in on-pump cardiac surgery. Gen. Thorac. Cardiovasc. Surg..

